# Suppression of CD300A inhibits the growth of diffuse large B-cell lymphoma

**DOI:** 10.18632/oncotarget.5152

**Published:** 2015-09-02

**Authors:** Lei Jiang, Yulian Xu, Xinli Zeng, Jianchen Fang, Herbert C. Morse, Jeff X. Zhou

**Affiliations:** ^1^ Department of Pathology, Zhejiang Provincial Key Laboratory of Pathophysiology, Ningbo University School of Medicine, Ningbo, China; ^2^ Department of ENT, Ningbo Second People's Hospital, Ningbo, China; ^3^ Ningbo Pathology Service Center, Ningbo, China; ^4^ Laboratory of Immunopathology, National Institute of Allergy and Infectious Diseases, National Institutes of Health, Rockville, MD, USA

**Keywords:** diffuse large B-cell lymphoma, CD300A, PI3K/AKT, lymphomagenesis

## Abstract

CD300A is a type I transmembrane receptor protein which has shown inhibitory effects on B-cell receptor-mediated signals. In an analysis of public dataset, we found that CD300A mRNA levels were inversely correlated with the overall survival time of patients with diffuse large B-cell lymphoma (DLBCL). To decipher the role of CD300A in DLBCL, we knocked down the expression levels of CD300A in DLBCL cells and found that decreasing levels of CD300A significantly inhibited cell proliferation of OCI-Ly01, Farage, and SUDHL-4 cells, but not of VAL, OCI-Ly10, or SUDHL-8 cells. Mechanistically, reduced expression of CD300A resulted in a marked attenuation of AKT phosphorylation, a key molecular event in tumorigenesis, in OCI-Ly01, Farage, and SUDHL-4 cells. Pharmacologic inhibition of PI3K displayed a similar inhibitory effect on cell proliferation. Furthermore, using a xenograft animal model, we found that decreasing levels of CD300A in OCI-Ly01 and Farage cells significantly inhibited tumor formation *in vivo*. Collectively, our results suggested an oncogenic role of CD300A in DLBCL which could serve as a potential biomarker and therapeutic target for this malignant disease.

## INTRODUCTION

Diffuse large B-cell lymphoma (DLBCL) is one of the most common non-Hodgkin lymphoma (NHL) in adults. Comparing to other types of NHL, DLBCL exhibits a high degree of heterogeneity [1–4]. Based on gene expression profile, DLBCL can be categorized into germinal center B-cell-like (GCB) and activated B-cell-like (ABC) subtypes. Patients with different molecular subtypes of DLBCL have distinct chemo-responsiveness and prognosis [5, 6]. Although the addition of monoclonal antibody drug rituximab to the standard chemotherapy regimen (cyclophosphamide, doxorubicin, vincristine, and prednisone) (R-CHOP) makes a subset of DLBCL curable, 50% of patients are refractory to the regimen [2, 7]. One of the possible mechanisms for the poor treatment response is the aberrantly constitutive activation of phosphatidylinositol 3-kinase (PI3K)-AKT pathway [8]. In a murine model, aberrantly active AKT promoted lymphomagenesis and drug resistance, which was reversible by the removal of AKT signaling [9]. In humans, DLBCL patients with high levels of phosphorylated AKT (*p*-AKT) in tumor tissues experienced a miserable survival time following treatment with CHOP [10]. In spite of the importance of AKT signaling in DLBCL, a paucity of information is available regarding the upstream mechanism responsible for PI3K-AKT activation. In addition to AKT, other driver mutations such as PTEN loss and CD79 mutations have also been identified in DLBCL [11, 12]. Thorough understanding of molecular pathology of DLBCL is crucial for the effective management of this malignancy.

A number of studies have shown that CD300A (IRp60), a type I transmembrane receptor of the CD300 family, acts as an inhibitory receptor in both myeloid and lymphoid cells [13]. In neutrophils, CD300A was able to inhibit CD32a (FcγRIIa)-mediated reactive oxygen species (ROS) production [14]. CD300A was also shown to inhibit IgE-mediated anaphylactic degranulation in basophils [15], and to suppress the effects of IL-5, GM-CSF, and eotaxin on human peripheral blood eosinophils [16]. In mast cells, cross-linking of CD300A with anti-IgE antibody suppressed stem cell factor-mediated survival and IgE-dependent degranulation by recruiting phosphatase SHP-1 and SHIP [17, 18]. In a murine model of allergic peritonitis, neutralization of CD300A augmented mast cell activation and subsequently eosinophil infiltration [17]. In addition, the inhibitory activities of CD300A were reported in monocytes [19, 20] and plasmacytoid dendritic cells [21], where activation of CD300A decreased Toll-like receptor 9 (TLR-9)-induced pro-inflammatory mediator expression.

In lymphoid lineage, CD300A was firstly discovered in natural killer (NK) cells. It was reported that CD300A exhibited strong inhibitions on NK cells' spontaneous cytotoxicity and NK-mediated cytolytic activity [22]. The inhibitory effects of CD300A on NK cell activity were critically dependent on the phosphorylation of tyrosine 267 located in the third immunoreceptor tyrosine-based inhibitory motif (ITIM) of CD300A [23]. Moreover, CD300A was capable of binding to tumor cells through interaction with phosphatidylserine (PS) in tumor cells, and blocking CD300A-PS interaction resulted in enhanced NK cell killing of tumor cell [24]. In CD4^+^ T cells, triggering of CD300A by a specific monoclonal antibody reduced T-cell receptor (TCR)-mediated Ca^2+^ flux [25]. Similarly, in human mature B cells, CD300A was found to act as a negative regulator in B-cell receptor (BCR)-mediated signaling such as Ca^2+^ mobilization, and decreasing levels of CD300A promoted B cell proliferation [26]. Interestingly, in HIV-infected patients, the expression levels of CD300A in B cells were significantly lower than that in healthy individuals, suggesting a role of CD300A in B-cell abnormality in HIV infection [26]. It was reported that CD300A expression was negative in most of Burkitt lymphoma cell lines, but positive in a DLBCL cell line SUDHL-5 [26]. However, little information was available regarding the role of CD300A in human DLBCL.

In an analysis of public available DLBCL gene expression datasets, we found that the levels of CD300A mRNA in DLBCL tumors were significantly inversely correlated with the overall survival time of DLBCL patients. This prompted us to examine the role of CD300A in DLBCL. In the present study, we found that the levels of CD300A in lymphoid tissues were greater in patients with DLBCL than that in patients with benign diseases. Decreasing CD300A expression in DLBCL cells significantly inhibited cell proliferation *in vitro* and suppressed tumor growth in a xenograft mouse model *in vivo*. The data suggested that CD300A may play an oncogenic role in DLBCL.

## RESULTS

### CD300A was expressed in primary patient samples and DLBCL cell lines

**Figure 1:**
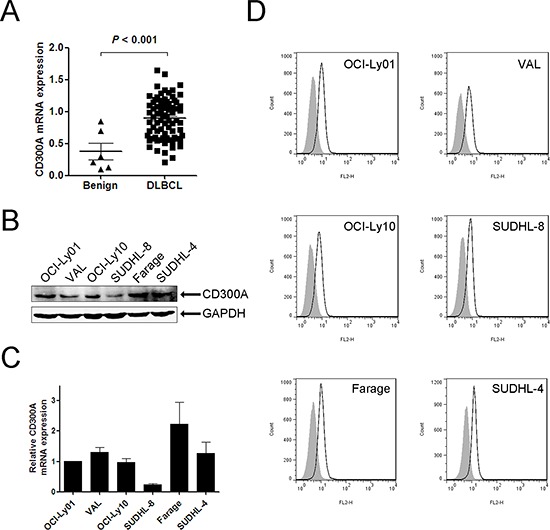
CD300A was expressed in DLBCL primary tissues and a panel of cell lines **A.** qPCR analysis of CD300A mRNA levels in benign lymphoid tissues (*n* = 6) and DLBCL tissues (*n* = 78). **B.** and **C.** CD300A mRNA and protein expression levels in six DLBCL cell lines (OCI-Ly01, VAL, OCI-Ly10, SUDHL-8, Farage, and SUDHL-4) measured using qPCR and western blot, respectively. Data were represented as mean ± SEM from at least three independent experiments. **D.** Flow cytometry evaluation of CD300A expression in DLBCL cells. Empty histogram represents binding of anti-CD300A mAb and gray histogram the binding of isotype control Ig.

To investigate the role of CD300A in the pathogenesis of DLBCL, we first examined CD300A expression in primary patient samples and a panel of DLBCL cell lines. Quantitative PCR data revealed that the mRNA levels of CD300A were significantly greater in DLBCL tumor tissues than that in human benign lymphoid tissues (Figure 1A). In DLBCL cell lines, CD300A was expressed in OCI-Ly01, VAL, OCI-Ly10, SUDHL-8, Farage, and SUDHL-4 cells at mRNA and protein levels (Figures 1B and 1C). In addition, CD300A was also found to be expressed on the surface of all these cell lines as analyzed using flow cytometry (Figure 1D).

### Downregulation of CD300A decreased DLBCL cell proliferation

**Figure 2:**
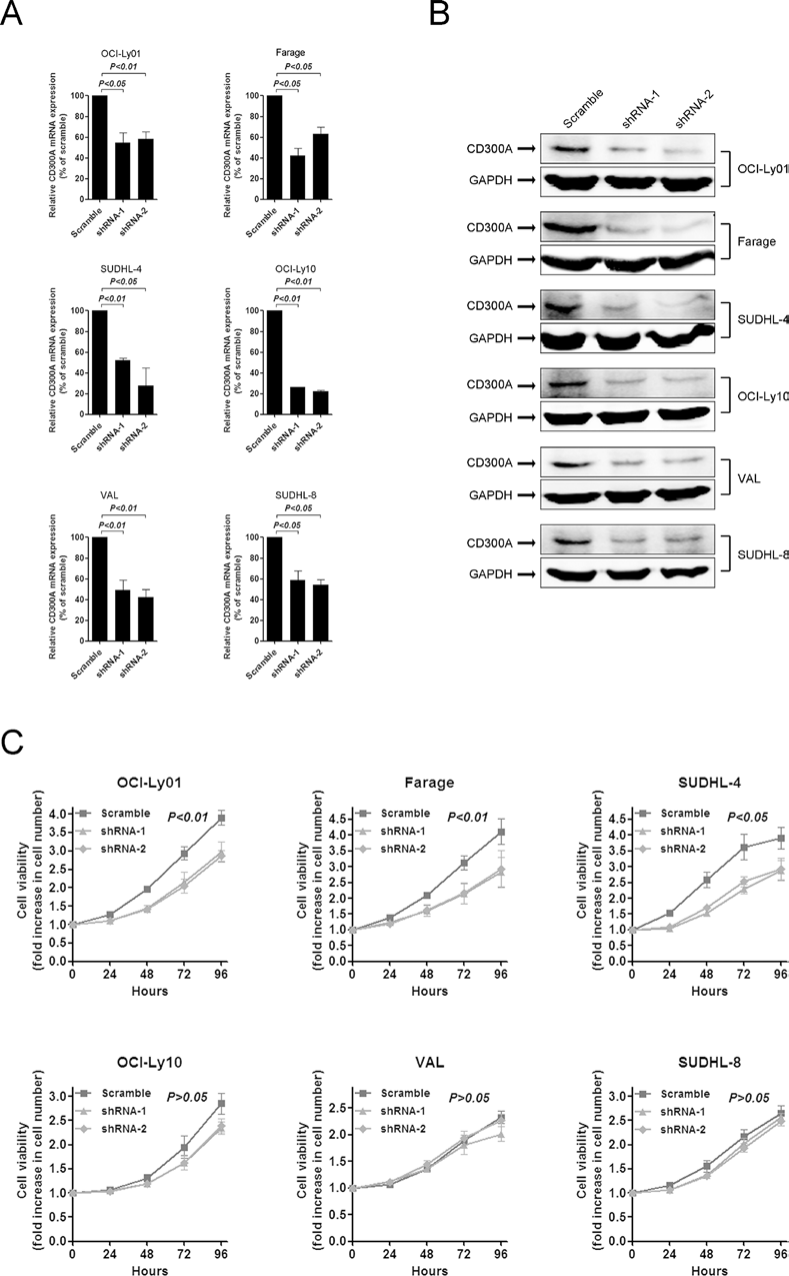
Inhibition of DLBCL cell proliferation by knockdown of CD300A levels **A.** and **B.** shRNAs-mediated knockdown of CD300A in DLBCL cells at both the mRNA (A) and protein (B) levels. **C.** Cell proliferation rate was detected at indicated times using MTS assay for DLBCL cells with or without CD300A knockdown. The fold increase in cell number indicates OD values relative to those at time 0. Data were represented as mean ± SEM from at least three independent experiments.

To determine the function of CD300A in DLBCL, we analyzed the proliferation rate of DLBCL cells with reduced levels of CD300A mediated by RNA interference. Two shRNAs (shRNA-1 and shRNA-2) specifically targeting CD300A were chosen and both significantly decreased CD300A expression levels in DLBCL cells at both mRNA and protein levels (Figures 2A and 2B). Decreasing levels of CD300A resulted in a significant inhibition of cell proliferation in OCI-Ly01, Farage, and SUDHL-4 cells, but not in VAL, OCI-Ly10, or SUDHL-8 cells (Figure 2C).

### Reducing levels of CD300A did not induce apoptosis in DLBCL cells

**Figure 3:**
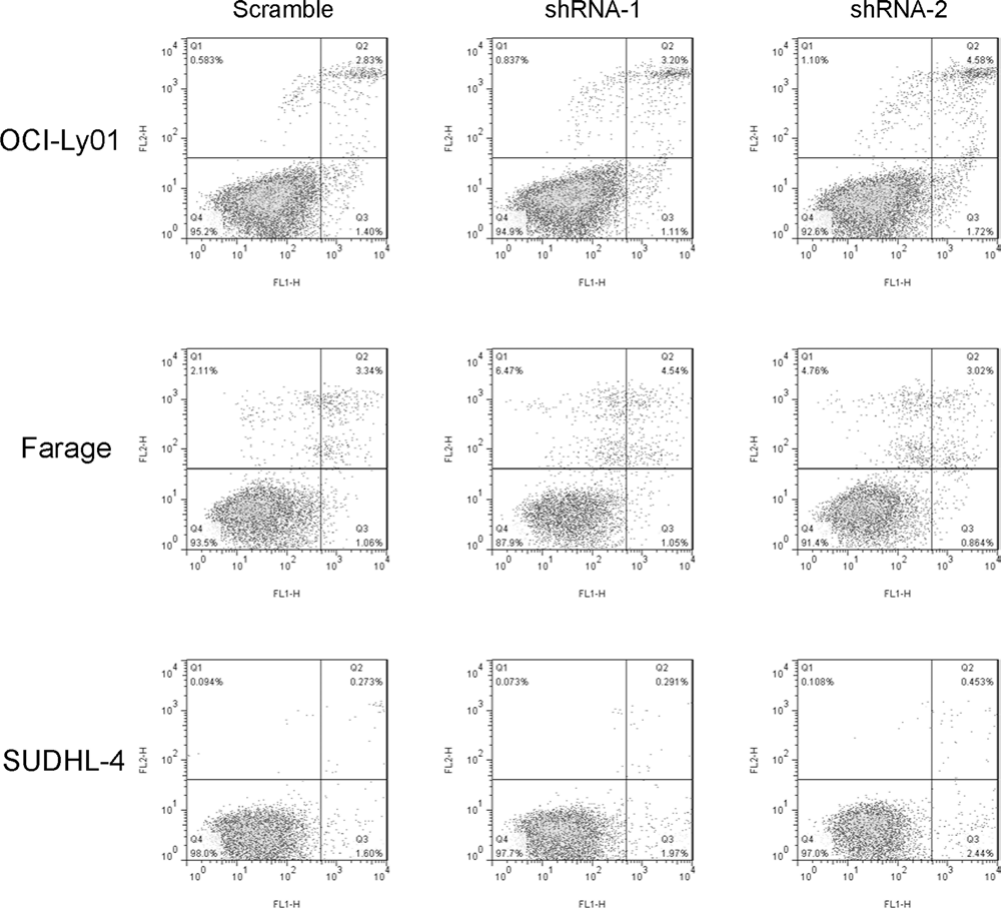
Reducing levels of CD300A did not induce apoptosis in DLBCL cells OCI-Ly01, Farage, and SUDHL-4 cells with or without CD300A knockdown were stained with FITC-conjugated annexin V and PE-conjugated PI. The percentage of apoptotic cells was analyzed using flow cytometry.

The assay used for assessing cell proliferation rate in this study measured total cell number after a given period of incubation time. Therefore, the decreased cell number following incubation of DLBCL cells with CD300A knocked-down could be due to an increased rate of cell death. To evaluate whether CD300A knockdown did induce apoptosis, CD300A-knockdown sensitive DLBCL cells were assayed using annexin V/PI dual staining. As shown in Figure 3, reducing levels of CD300A did not induce significant apoptosis in OCI-Ly01, Farage, and SUDHL-4 cells.

### CD300A knockdown decreased the rate of DLBCL cell division

**Figure 4:**
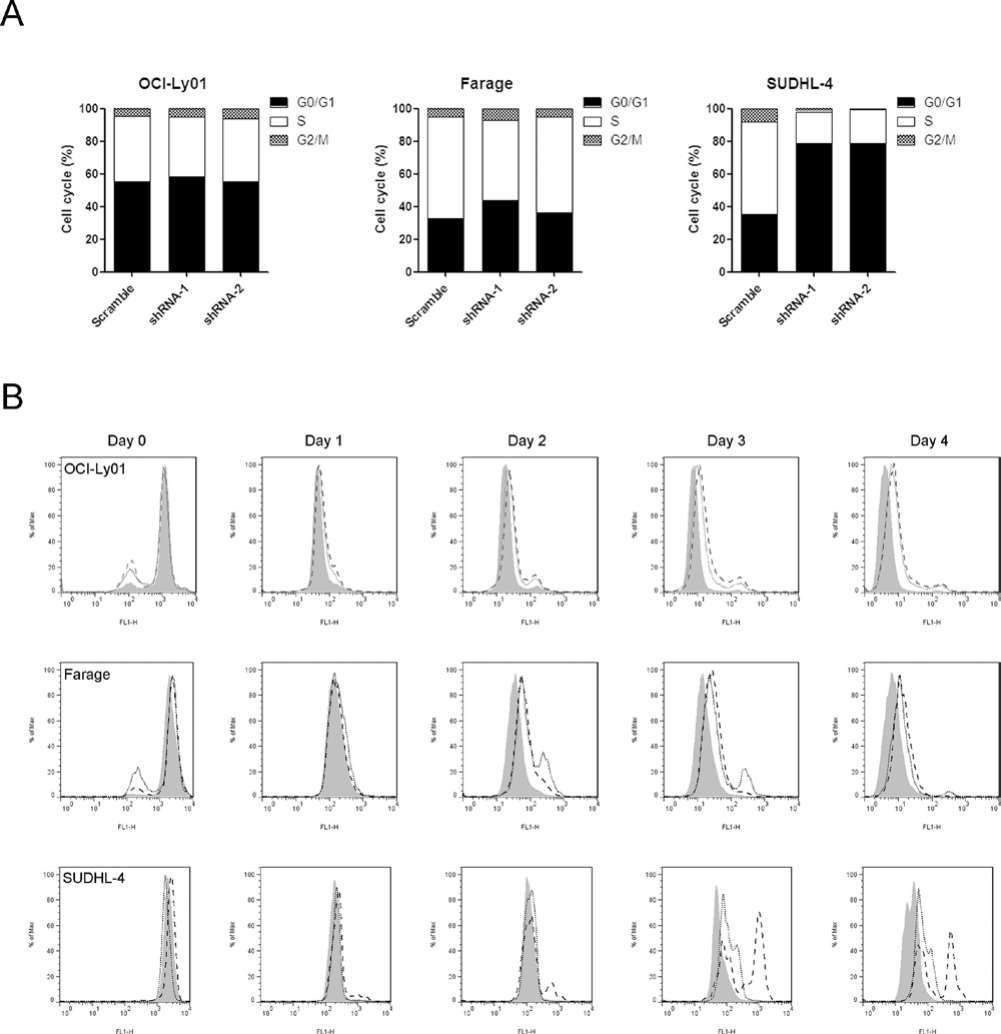
CD300A knockdown decreased the rate of DLBCL cell division **A.** Cell cycle distribution of OCI-Ly01, Farage, and SUDHL-4 cells with or without CD300A knockdown was assessed by measuring DNA content of PI-stained cells using flow cytometry. **B.** The cell division was measured using CFSE labeling method. Following CFSE incorporation into the cells, the dilution of fluorescence intensity was determined every 24 h using flow cytometry.

To identify the mechanisms underlying the decreased proliferation of DLBCL cells with CD300A knocked-down, we analyzed cell cycle of OCI-Ly01, Farage, and SUDHL-4 cells. To our surprise, reducing levels of CD300A selectively increased the percentage of cells in G1 phase, decreased the percentage of cells in S and G2 phases in SUDHL-4 cells, but had no significant effects on OCI-Ly01 or Farage cells (Figure 4A). We subsequently examined cell division by labeling cells with CFSE and tracking cellular fluorescence intensity dilution at 0, 24, 48, 72, and 96 h. Consistent with cell proliferation tests, knockdown of CD300A substantially decreased the division rate of OCI-Ly01, Farage, and SUDHL-4 cells (Figure 4B). These results indicated that the decreased proliferation rate of DLBCL cells caused by CD300A knockdown was due to a slowed-down cell division and/or cell cycle arrest.

### PI3K/AKT activation was inhibited in CD300A-knockdown DLBCL cells

**Figure 5:**
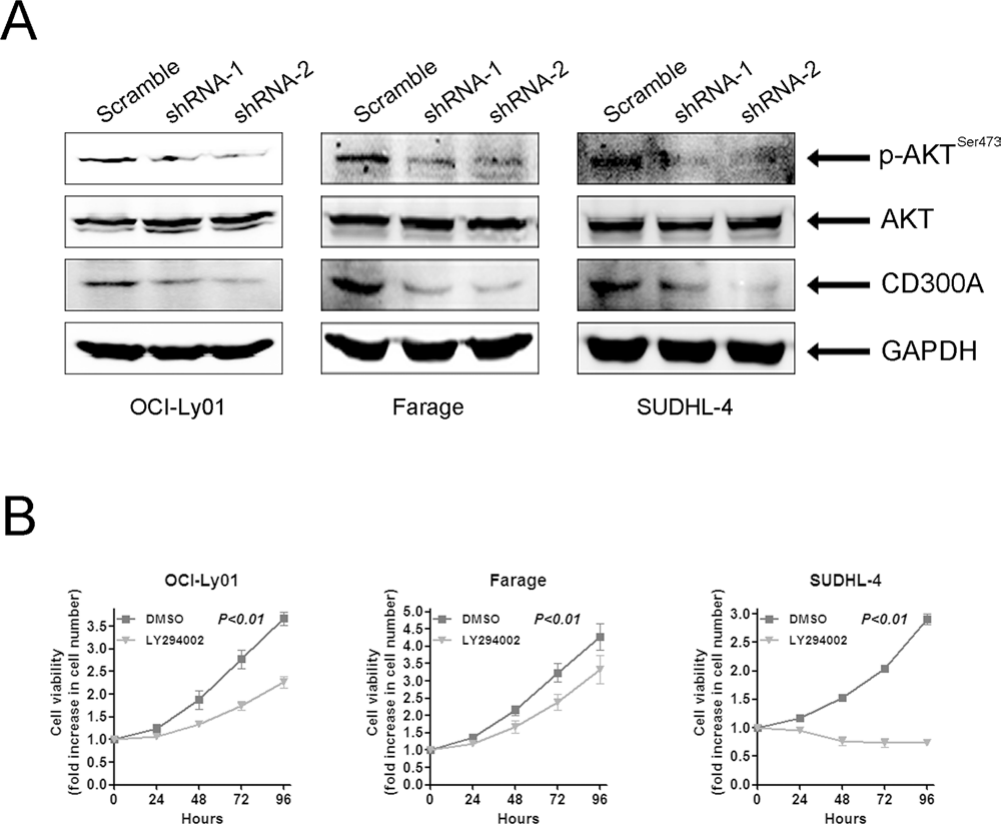
CD300A knockdown suppressed AKT phosphorylation in DLBCL cells **A.** Whole cell lysates obtained from OCI-Ly01, Farage, and SUDHL-4 cells with or without CD300A knockdown were analyzed using western blotting with antibodies specific for phospho-AKT (SER473), total AKT, CD300A, and GAPDH. **B.** Cell proliferation assay of OCI-Ly01, Farage, and SUDHL-4 cells treated with LY294002 (5 μM). The fold increase in cell number indicates OD values relative to those at time 0. Data were represented as mean ± SEM from at least three independent experiments.

Previous work suggested that the PI3K/AKT pathway was constitutively activated and played a critical role in mediating survival and growth in DLBCL cells [8, 27]. To determine whether PI3K signaling pathway was involved in the effect of CD300A, we tested AKT phosphorylation which was a direct downstream target of PI3K activation. The data showed that the levels of phosphorylated AKT (*p*-AKT) were significantly decreased in OCI-Ly01, Farage, and SUDHL-4 cells with CD300A knocked-down (Figure 5A). In the meantime, we used LY294002, a chemical specifically inhibiting AKT phosphorylation, to treat DLBCL cells and found that suppression of AKT activity led to a significant inhibition of cell proliferation in OCI-Ly01, Farage, and SUDHL-4 cells (Figure 5B), but not in VAL, OCI-Ly10, or SUDHL-8 cells (data not shown). Therefore, these results suggested that CD300A knockdown was selectively toxic to PI3K inhibition-sensitive DLBCL cells, and PI3K/AKT pathway may play a role in mediating CD300A's effect on cell growth.

### Decreasing levels of CD300A inhibited DLBCL growth *in vivo*

**Figure 6:**
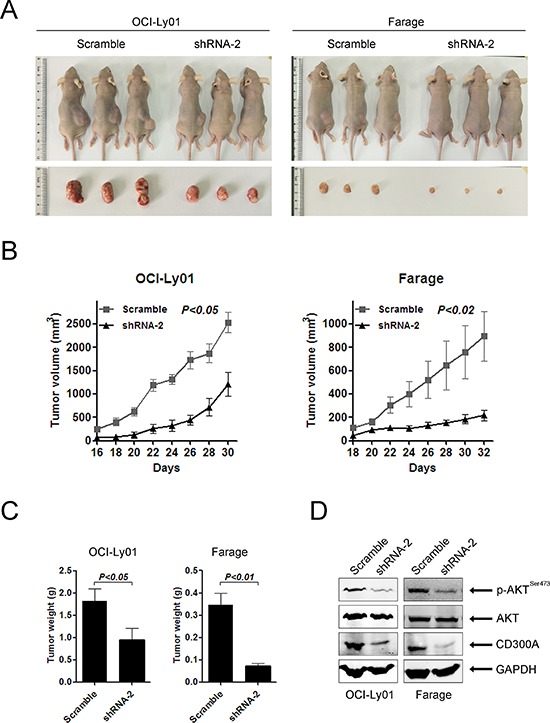
Loss of CD300A suppressed DLBCL tumor growth *in vivo* **A.** Representative photographs of mice 30–32 days after separately injected in the flank with OCI-Ly01 and Farage cells with or without CD300A knockdown (*n* = 5 for each group). **B.** The increase in tumor volumes in mice received OCI-Ly01 and Farage cells with or without CD300A knockdown. Tumor volumes were measured every two days. **C.** The tumor weights of mice received OCI-Ly01 and Farage cells with or without CD300A knockdown. The tumors were weighted on day 30–32 when all animals were sacrificed. Data were represented as mean ± SEM from five mice in each group. **D.** The decreased levels of *p*-AKT in tumors received OCI-Ly01 and Farage cells with CD300A knockdown in comparison to those received control cells. The levels of specific proteins were analyzed using western blotting.

To evaluate the role of CD300A in DLBCL development *in vivo*, we separately engrafted OCI-Ly01 and Farage cells expressing shRNAs specifically targeting CD300A into nude mice. The results showed that downregulation of CD300A led to a profound suppression of tumor growth, as evidenced by a significant reduction in tumor volume and weight (Figures 6A, 6B, and 6C). Furthermore, western blot analysis of tumor tissues isolated from the mice indicated decreasing levels of *p*-AKT in tissues with CD300A knockdown (Figure 6D).

## DISCUSSION

Aberrant activity of PI3K signaling pathway plays a key role in cell proliferation in DLBCL by activating AKT and PDK1 [8, 12]. However, very limited information has been available regarding the upstream molecular mechanisms causing constitutive PI3K activation. In the present study, we provided molecular evidences that CD300A was a regulator of AKT phosphorylation, and loss of CD300A resulted in suppression of AKT phosphorylation and DLBCL cell growth both *in vitro* and *in vivo*.

The expression and function of CD300A in myeloid cells have been well characterized. The expression of CD300A in lymphoid cells, however, remained to be controversial. In an early report in 1993, Daish et al found that a subset of peripheral blood B cells was able to react with CMRF-35 monoclonal antibody which recognized both CD300A and CD300C [28]. On the contrary, Clark et al showed negative CD300A expression in B cells [29]. A recent study reported that the expression levels of CD300A were variable during different development stages of human B cells [26]. In human peripheral blood and tonsil B cells, Borrego and colleagues showed that only memory B cells had significant levels of CD300A, but naïve and germinal center B cells CD300A had negligible amount of CD300A [26]. In malignant B cells, CD300A was found to be positive in an ABC subtype DLBCL cell line SUDHL-5 [26]. The data from our current study indicated that CD300A was positive in DLBCL cells including OCI-Ly01, VAL, OCI-Ly10, SUDHL-8, Farage, and SUDHL-4. It seemed that there was no apparent correlation between the levels of CD300A and DLBCL molecular subtypes. Importantly, we found that the levels of CD300A in DLBCL tumor tissues were significant greater than that in lymphoid tissues from benign diseases, suggesting a potential clinical significance of CD300A for DLBCL.

In previous studies, the results that CD300A ligation inhibited Ca^2+^ mobilization and decreasing CD300A levels promoted cell proliferation pointed to an inhibitory role of CD300A in human B cells [26]. Nevertheless, there were also evidences showing that CD300A possessed an activating role as its engagement increased IFN- α secretion by human plasmacytoid dendritic cells [21]. Similarly, our present study suggested an oncogenic role of CD300A in B-cell lymphoma in which loss of CD300A suppressed DLBCL growth both *in vitro* and *in vivo*. Taken together, our and other's data suggested an important function of CD300A both physiologically and pathologically in B lymphocytes.

Similar to the effects of SYK inhibition on DLBCL cells [30], we found that only OCI-Ly01, Farage, and SUDHL-4 cells, but not the other three types of DLBCL cell lines, were sensitive to the knockdown of CD300A. Because CD300A was an upstream molecule regulating PI3K/AKT activation, the insensitiveness of OCI-Ly10, SUDHL-8, and VAL cells to CD300A knockdown might be due to their resistance to PI3K inhibition as reported previously. It was shown that for SUDHL-8 cells, LY294002 treatment did not affect AKT phosphorylation and apoptosis, suggesting that SUDHL-8 cells were refractory to PI3K inhibition [27]. Although LY294002 and 15e (a PI3K inhibitor potently inhibits p100α activity) blocked AKT phosphorylation in most ABC subtype of DLBCL cells, both treatment had minimal effects on the growth, division, and apoptosis of OCI-Ly10 and some other ABC DLBCL cells except for HBL-1 and TMD8 cells [8]. These results might be related to the fact that HBL-1 and TMD8 cells harbored CD79B mutation, whereas OCI-Ly10 and other ABC DLBCL cells had wild-type CD79B [11]. Additionally, as an important downstream target of AKT, mTOR played a crucial role in DLBCL [10]. VAL cells were particularly resistant to the mTOR inhibitor due to the lack of eukaryotic initiation factor 4E binding protein-1 that was a key negative regulator of transduction controlled by mTOR [31]. Furthermore, studies using gene expression profiling identified that OCI-Ly01 cells were sensitive but SUDHL-8 cells were resistant to rapamycin [32]. All these evidences combined with our data helped explain why OCI-Ly01, Farage, and SUDHL-4 cells were sensitive but other three types of DLBCL cells were insensitive to CD300A knockdown as found in the present study.

Collectively, our results clearly identified the high expression of CD300A in DLBCL tumor tissues and a novel oncogenic role of CD300A in DLBCL. The loss of CD300A inhibited DLBCL cell proliferation *in vitro* and suppressed tumor growth *in vivo*, an effect likely mediated by its inhibition on PI3K/AKT activation. Further studies would be performed to elucidate the clinical significance of CD300A in DLBCL. Because CD300A is a cell-surface molecule which can be conveniently targeted by small molecules or antibodies, our study presented a potential therapeutic target for DLBCL treatment.

## MATERIALS AND METHODS

### Human DLBCL tissues and benign tissues

Formalin-fixed paraffin-embedded (FFPE) human DLBCL tissues from randomly and anonymously selected patients were provided by Ningbo Pathology Service Center (NPSC) with the approval of Ethics Committee of Human Tissue Use of NPSC. Human non-neoplasm lymphoid tissues (tonsils) were obtained from patients with tonsillitis who received tonsillectomy at Ningbo Second People's Hospital with written informed consents of the patients and the approval of Research Ethics Committee of the hospital. The pathological diagnosis for all tissue samples were verified by a hematology pathologist (JF).

### Cell culture, retroviral construct and transduction

Human DLBCL cell lines OCI-Ly01, VAL, OCI-Ly10, SUDHL-8, Farage, and SUDHL-4 cells were purchased from ATCC (Shanghai, China) and cultured in RPMI-1640 medium (Hyclone, Utah, USA) supplemented with 10% fetal bovine serum (FBS) (Ausgenex, Australia), 100 U/mL penicillin, 100 U/mL streptomycin, and 2 mM L-glutamine. Human embryonic kidney 293T cells were maintained in Dulbecco modified Eagle media (DMEM; Hyclone) with 10% FBS. Cells were cultured at 37°C in a 5% CO_2_ humidified incubator.

Lentiviral plasmids containing optimized CD300A short hairpin (shRNA) or scramble sequences were produced using 293T cells, and were used to infect DLBCL cells as described [4, 33]. The sequences for CD300A shRNAs used in the present study included shRNA-1 (5′-GATGTTTCAGAAATGGATCAA-3′), shRNA-2 (5′-CCCAGGGAAGAACTTCACTAT-3′), and a scramble shRNA (5′-CCTAAGGTTAAGTCGCCCTCG-3′).

The surface expression of CD300A in lymphoma cells was determined using a phycoerythrin (PE)-conjugated antibody against human CD300A (clone E59.126, Beckman Coulter, Marseille, France). After staining for 30 min on ice, cells were resuspended in phosphate-buffered saline (PBS) and analyzed using a FACS Calibur instrument (Becton Dickinson, CA, USA). The isotypic antibody (clone 679.1Mc7, Beckman Coulter) was used as control.

### Quantitative real-time PCR (qRT-PCR)

The levels of *CD300A* mRNA was determined using qRT-PCR as previously described [26]. Briefly, total RNA was extracted using Trizol reagent (Invitrogen, Shanghai, China) according to the manufacturer's instructions. cDNA was prepared using a reverse transcription kit (Thermo Scientific, Shanghai, China). qRT-PCR was performed using SYBR Green PCR master mix on a LightCycler 480 system (Roche, Shanghai, China). All samples were run in triplicate. The levels of *CD300A* expression were normalized to that of glyceraldehyde-3-phosphate dehydrogenase (*GAPDH*). The primers used for *CD300A* were 5′-GCTGAGAAAGACGAGAGACC-3′ (forward) and 5′-TTCATCATCTTCCACCAGTG-3′ (reverse), and for *GAPDH* were 5′-CGACCACTTTGTCAAGCTCA-3′ (forward) and 5′-CCCTGTTGCTGTAGCCAAAT-3′ (reverse).

### Western blotting analysis

Cellular proteins were separated using 10% SDS-polyacrylamide gel, electrotransferred onto a polyvinylidene difluoride membrane, and incubated with specific antibodies. The CD300A antibody was purchased from Santa Cruz Biotechnology (CA, USA). Antibodies against GAPDH, AKT, and phosphor-AKT-Ser473 were purchased from Cell Signaling Technology (Shanghai, China). After incubation with the corresponding secondary antibodies, membranes were incubated with goat anti-rabbit IR Dye 800CW or goat anti-mouse IR Dye 680 (LI-COR Biosciences, Lincoln, NE, USA), washed, and scanned for densitometry using an Odyssey infrared imaging system (LI-COR Biosciences, CA, USA).

### Cell viability assay

Cells were plated in a 96-well plate (20,000 cells/well) in triplicates. Cell viability was determined using MTS assay (Promega, WI, USA) at 0, 24, 48, 72, and 96 h after seeding. The absorbance was read at 490 nm using a spectrophotometer.

### Cell division and cell cycle analyses

Cell division (cell proliferation) was analyzed by labeling cells with carboxyfluorescein diacetate succinimidyl ester (CFSE) (Sigma-Aldrich, MO, USA) as described previously [34]. Briefly, cells were incubated with CFSE for 10 min at 37°C in the dark, washed, and continued incubation for additional 4 d. Cell division was examined at day 1, 2, 3, and 4 using a FACS instrument. For cell cycle analysis, cells were washed with PBS and fixed in ethanol for 30 min at 4°C, and incubated with propidium iodide (PI) and RNase (Sigma-Aldrich) for 30 min at 37°C in the dark and subjected FACS analysis.

### Apoptosis analysis

Cells were washed with PBS and fixed in ethanol for 30 min at 4°C, and incubated with Annexin V and PI using a commercially kit (V13241, Invitrogen, Shanghai, China) following the manufacturer's recommendation.

### Xenograft animal model

The animal use protocol was approved by the Animal Care Committee of Ningbo University (Protocol number 2014006). Five-week old male BALB/c nude mice (Shanghai laboratory animal center, Shanghai, China) were kept in a clean environment. OCI-Ly01 and Farage cells stably expressing CD300A shRNA-2 or scramble shRNA were separately injected subcutaneously into the right flank of mice. Tumor's maximal length and width were measured every two days using a digital caliper, and tumor volume (V) was calculated using the following formula: *V* = (length × width^2^)/2. Mice were sacrificed at day 30–32 following tumor-cell implantation. The body weight of tumor-bearing animals did not change significantly during the study course. The tumors were excised and weighted. Each tumor tissue was homogenized for the preparation of protein samples for western blotting analysis.

### Statistical analysis

Data were expressed as mean ± SEM. The differences between experimental groups were examined using Fisher's exact text or Student *t* test where appropriate using SPSS software (Chicago, IL, USA), and was deemed to be statistically significant when *P* < 0.05.
