# Correction: Suppression of CD300A inhibits the growth of diffuse large B-cell lymphoma

**DOI:** 10.18632/oncotarget.17684

**Published:** 2017-05-08

**Authors:** Lei Jiang, Yulian Xu, Xinli Zeng, Jianchen Fang, Herbert C. Morse III, Jeff X. Zhou

**Present**: Due to an error during manuscript preparation, the primer sequences for CD300A included in the published paper are incorrect.

**Correct:** The proper primer sequences are shown below. The authors sincerely apologize for this oversight.

Original article: Oncotarget. 2015; 6:31191-202. doi: 10.18632/oncotarget.5152

## QUANTITATIVE REAL-TIME PCR (QRT-PCR)

The levels of CD300A mRNA was determined using qRT-PCR as previously described [26]. Briefly, total RNA was extracted using Trizol reagent (Invitrogen, Shanghai, China) according to the manufacturer’s instructions. cDNA was prepared using a reverse transcription kit (Thermo Scientific, Shanghai, China). qRT-PCR was performed using SYBR Green PCR master mix on a LightCycler 480 system (Roche, Shanghai, China). All samples were run in triplicate. The levels of CD300A expression were normalized to that of glyceraldehyde-3-phosphate dehydrogenase (GAPDH). The primers used for CD300A were 5′-GGTCCCAGCATCAACGTCAA-3′ (forward) and 5′-CCCACTGCAAACAGGGTAGT-3′ (reverse), and for GAPDH were 5′-CGACCACTTTGTCAAGCTCA-3′ (forward) and 5′-CCCTGTTGCTGTAGCCAAAT-3′ (reverse).

